# A Nation-Wide, Multi-Center Study on the Quality of Life of ALS Patients in Germany

**DOI:** 10.3390/brainsci11030372

**Published:** 2021-03-14

**Authors:** Tara Peseschkian, Isabell Cordts, René Günther, Benjamin Stolte, Daniel Zeller, Carsten Schröter, Ute Weyen, Martin Regensburger, Joachim Wolf, Ilka Schneider, Andreas Hermann, Moritz Metelmann, Zacharias Kohl, Ralf A. Linker, Jan Christoph Koch, Boriana Büchner, Ulrike Weiland, Erik Schönfelder, Felix Heinrich, Alma Osmanovic, Thomas Klopstock, Johannes Dorst, Albert C. Ludolph, Matthias Boentert, Tim Hagenacker, Marcus Deschauer, Paul Lingor, Susanne Petri, Olivia Schreiber-Katz

**Affiliations:** 1 Department of Neurology, Hannover Medical School, 30625 Hannover, Germany; Peseschkian.Tara@mh-hannover.de (T.P.); Erik.Schoenfelder@stud.mh-hannover.de (E.S.); Felix.Heinrich@stud.mh-hannover.de (F.H.); dr.almaosmanovic@gmail.com (A.O.); Petri.Susanne@mh-hannover.de (S.P.); 2 Department of Neurology, Klinikum Rechts der Isar, Technical University of Munich, 81675 Munich, Germany; isabell.cordts@tum.de (I.C.); marcus.deschauer@mri.tum.de (M.D.); paul.lingor@tum.de (P.L.); 3 Department of Neurology, University Hospital Carl Gustav Carus, 01307 Dresden, Germany; Rene.Guenther@uniklinikum-dresden.de; 4 German Center for Neurodegenerative Diseases (DZNE), 01307 Dresden, Germany; 5 Department of Neurology, University Medicine Essen, 45147 Essen, Germany; benjamin.stolte@uk-essen.de (B.S.); tim.hagenacker@uk-essen.de (T.H.); 6 Department of Neurology, University of Würzburg, 97080 Würzburg, Germany; Zeller_D@ukw.de; 7 Hoher Meißner Clinic, Neurology, 37242 Bad Sooden-Allendorf, Germany; Schroeter@reha-klinik.de; 8 Department of Neurology, Ruhr-University Bochum, BG-Kliniken Bergmannsheil, 44789 Bochum, Germany; ute.weyen@bergmannsheil.de; 9 Department of Molecular Neurology, Friedrich-Alexander-University Erlangen-Nürnberg, 91054 Erlangen, Germany; Martin.Regensburger@uk-erlangen.de; 10 Department of Neurology, Diakonissen Hospital Mannheim, 68163 Mannheim, Germany; j.wolf@diako-mannheim.de; 11 Department of Neurology, Martin-Luther University Halle/Saale, 06120 Halle, Germany; Ilka.Schneider@sanktgeorg.de; 12 Department of Neurology, Klinikum Sankt Georg, 04129 Leipzig, Germany; 13 Translational Neurodegeneration Section “Albrecht-Kossel”, Department of Neurology, University Medical Center Rostock, University of Rostock, 18147 Rostock, Germany; Andreas.Hermann@med.uni-rostock.de; 14 German Center for Neurodegenerative Diseases Rostock/Greifswald, 18147 Rostock, Germany; 15 Department of Neurology, University Hospital Leipzig, 04103 Leipzig, Germany; Moritz.Metelmann@medizin.uni-leipzig.de; 16 Department of Neurology, University of Regensburg, 93053 Regensburg, Germany; zacharias.kohl@klinik.uni-regensburg.de (Z.K.); Ralf.Linker@klinik.uni-regensburg.de (R.A.L.); 17 Department of Neurology, University Medicine Göttingen, 37075 Göttingen, Germany; jkoch@med.uni-goettingen.de; 18 Friedrich-Baur Institute, Department of Neurology, University Hospital, Ludwig Maximilian University of Munich, 80336 Munich, Germany; Boriana.Buechner@med.uni-muenchen.de (B.B.); Thomas.Klopstock@med.uni-muenchen.de (T.K.); 19 Department of Neurology, University of Ulm, 89081 Ulm, Germany; Ulrike.Weiland@uniklinik-ulm.de (U.W.); johannes.dorst@rku.de (J.D.); albert.ludolph@rku.de (A.C.L.); 20 Munich Cluster for Systems Neurology (SyNergy), 80336 Munich, Germany; 21 German Center for Neurodegenerative Diseases (DZNE), 80336 Munich, Germany; 22 German Center for Neurodegenerative Diseases (DZNE), 89081 Ulm, Germany; 23 Department of Neurology with the Institute of Translational Neurology, University Hospital Münster, 48149 Münster, Germany; Matthias.Boentert@ukmuenster.de; 24 Department of Medicine, UKM Marienhospital, 48565 Steinfurt, Germany

**Keywords:** Amyotrophic Lateral Sclerosis (ALS), Amyotrophic Lateral Sclerosis Assessment Questionnaire 5 (ALSAQ-5), ALS treatment, “bulbar-onset” ALS (*b*ALS), “limb-onset” ALS (*l*ALS), EuroQol Five Dimension Five Level Scale (EQ-5D-5L), health-related quality of life (HRQoL), quality of life (QoL), symptom-specific treatment, assistive devices

## Abstract

Improving quality of life (QoL) is central to amyotrophic lateral sclerosis (ALS) treatment. This Germany-wide, multicenter cross-sectional study analyses the impact of different symptom-specific treatments and ALS variants on QoL. Health-related QoL (HRQoL) in 325 ALS patients was assessed using the Amyotrophic Lateral Sclerosis Assessment Questionnaire 5 (ALSAQ-5) and EuroQol Five Dimension Five Level Scale (EQ-5D-5L), together with disease severity (captured by the revised ALS Functional Rating Scale (ALSFRS-R)) and the current care and therapies used by our cohort. At inclusion, the mean ALSAQ-5 total score was 56.93 (max. 100, best = 0) with a better QoL associated with a less severe disease status (β = −1.96 per increase of one point in the ALSFRS-R score, *p* < 0.001). “Limb-onset” ALS (*l*ALS) was associated with a better QoL than “bulbar-onset” ALS (*b*ALS) (mean ALSAQ-5 total score 55.46 versus 60.99, *p* = 0.040). Moreover, with the ALSFRS-R as a covariate, using a mobility aid (β = −7.60, *p* = 0.001), being tracheostomized (β = −14.80, *p* = 0.004) and using non-invasive ventilation (β = −5.71, *p* = 0.030) were associated with an improved QoL, compared to those at the same disease stage who did not use these aids. In contrast, antidepressant intake (β = 5.95, *p* = 0.007), and increasing age (β = 0.18, *p* = 0.023) were predictors of worse QoL. Our results showed that the ALSAQ-5 was better-suited for ALS patients than the EQ-5D-5L. Further, the early and symptom-specific clinical management and supply of assistive devices can significantly improve the individual HRQoL of ALS patients. Appropriate QoL questionnaires are needed to monitor the impact of treatment to provide the best possible and individualized care.

## 1. Introduction

Amyotrophic lateral sclerosis (ALS) is an incurable, mainly sporadic neurodegenerative disorder, which affects the upper and lower motor neurons (UMN and LMN) [1]. It is the most common adult-onset motor neuron disease (MND) with a current incidence in Germany of 3.1/100,000 and a prevalence of 8/100,000 [2]. It manifests predominantly between the age of 50 and 70 years and rapidly progresses, usually resulting in death, often due to respiratory failure, within 2–5 years after onset [1]. Depending on the localization of first symptoms, patients are classified as having either “limb-onset” (*l*ALS), accounting for two-thirds of all ALS cases in which spinal alpha motor neurons are affected first, or “bulbar-onset” (*b*ALS) ALS [3]. The most common symptoms of *l*ALS are muscle weakness, fasciculations and muscle cramps [4], while the predominant symptoms of *b*ALS include dysarthria and dysphagia [1,3].

As no specific curative interventions exist, improving patient care and quality of life (QoL) has become a priority in clinical management of ALS. As such, there is a need for interventions to be evaluated with regards to their individual impact so that patients can receive the best possible care. Additionally, such evaluations have a significant socioeconomic impact given that the lifetime cost of one ALS patient (from the first symptom to death) has been estimated to be €246,184 [5]. Interventions can be evaluated through analyzing patient QoL, adding weight to the importance of having robust and reliable QoL questionnaires.

QoL was defined by the World Health Organisation as a “broad ranging concept affected in a complex way by the person’s physical health, psychological state, level of independence, social relationships and their relationship to salient features of their environment” [6]. Health-related QoL (HRQoL) was introduced to provide a more disease-specific analysis, as it has been shown to decline in correlation to decreased mental or physical performance [7,8]. Factors that have commonly been found to negatively influence HRQoL in ALS patients include limited physical mobility [5,9,10], disease severity [5,9,10,11], depression [7,9,10,11] and mechanical ventilator use [10]. Besides the neuroprotective drug riluzole, which has been found to prolong life [1,12,13], interventions developed to alleviate symptoms of ALS include non-invasive ventilation [1,14,15], gastrostomy [1,16], communication aids [1,17,18,19], mobility aids [20] and anticholinergic drugs [1,21], among others. 

In assessing the impact of some of these interventions on QoL, previous studies have found, for example, that Nuedexta is an effective remedy for the symptom of uncontrollable laughing and crying [22], and that anticholinergic drugs are effective at countering excessive salivary secretion and muscle spasms [23]. Additionally, some communication devices, like eye tracking devices, for example Tobii Dynavox (Tobii Dynavox, Danderyd, Sweden) have been found to have a huge impact on QoL for patients who are being ventilated. They help maintain patient autonomy, involve them in the decision-making process, and enable them to answer more than just yes/no questions by head-nodding [24]. Furthermore, some studies have found spirituality and religion to also have a positive impact on QoL [25,26]. However, as interactions of and influencing factors on QoL are a very complex and multidimensional topic, we attempt to contribute an analysis of some of these symptom-specific treatments as possible influencers on QoL, based on the interventions used by the analyzed cohort, which is the largest patient cohort in Germany to date.

Therapeutic interventions differ depending on the ALS variant. While many studies have shown that *b*ALS patients show worse QoL and have a shorter survival [3,27,28,29,30,31], none specifically compared the difference in QoL between the different variants.

Numerous QoL questionnaires have been developed worldwide. These include the EuroQol Five Dimension Five Level Scale (EQ-5D-5L) [32], an HRQoL-focused questionnaire, which has been used to measure QoL in patients suffering from a variety of diseases, including narcolepsy [33], Parkinson’s disease [34], Alzheimer disease [35] and ALS [5,9]. Separately, the Amyotrophic Lateral Sclerosis Assessment Questionnaire 5 (ALSAQ-5) has been developed as a shorter version of the Amyotrophic Lateral Sclerosis Assessment Questionnaire 40 (ALSAQ-40) [36] in order to specifically investigate HRQoL in ALS. The ALSAQ-5 has not yet been validated in German. Thus, this paper aimed to show a correlation between the shorter ALSAQ-5 version and the widely used EQ-5D-5L and tries to fill another gap relating to a lack of comparative analyses between the two questionnaires.

To sum up, this study seeks to remedy the above-mentioned gaps by looking at the following aims:

1. Perform a descriptive analysis of HRQoL in a large, nation-wide ALS cohort, with an emphasis on the differences between *l*ALS and *b*ALS;
2. Perform an analysis of some medical interventions that may influence QoL in ALS patients treated according to current standards of care; and
3. Perform a comparison of two QoL questionnaires, namely the EQ-5D-5L and the ALSAQ-5.

## 2. Methods

### 2.1. Study Design, Setting and Participants

**Figure 1:**
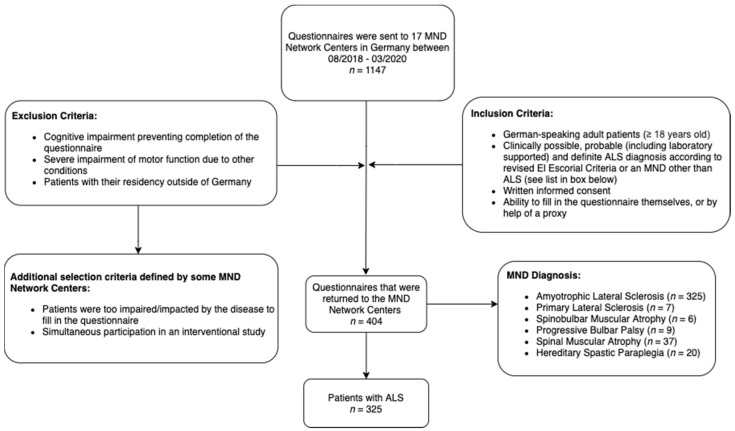
Flow chart diagram depicting identification of the final study cohort and inclusion and exclusion criteria. Abbreviations: ALS, Amyotrophic Lateral Sclerosis; MND, Motor Neuron Disease; *n*, number.

Data collection for this multi-center cross-sectional study took place between August 2018 and March 2020. The questionnaire was sent to 17 cooperating MND Network Centers across Germany [37] (Hannover; Munich (Technical University of Munich and Ludwig Maximilian University of Munich); Dresden; Würzburg; Bad Sooden-Allendorf; Bochum; Erlangen; Ulm; Mannheim; Halle-Wittenberg; Rostock; Leipzig; Regensburg; Göttingen; Münster and Essen). Patients were either screened for enrolment during their routine medical visits, or screening was based on their last recorded medical visit. In the latter case, the questionnaire was sent by mail. We do not expect that the method of patient screening would influence our results, as the patients filled in the questionnaire by themselves in both circumstances. Inclusion and exclusion criteria and the full data collection process is shown in Figure 1. In total, 325 ALS patients diagnosed with clinically possible, probable (including laboratory supported) and definite ALS, following the revised El Escorial criteria [38], were included in the statistical analysis. Patients diagnosed with an MND other than ALS were not analyzed for the purpose of this paper, but their data will be presented in further studies.

This study report was structured following the reporting guidelines to strengthening the reporting of observational studies in epidemiology (STROBE) [39]. 

### 2.2. Study Questionnaire

The patients answered a standardized, self-designed, pretested and paper-based questionnaire by hand, created as part of a study assessing the disease costs of ALS [5,40]. The first part of the questionnaire was an inventory of the patients’ demographics (diagnosis, gender, age, body mass index, marital status, state and type of health insurance) and disease history (first symptoms, age at disease manifestation and genetics). The second part addressed questions regarding impairment of daily activities in different domains (temporal, physical, psychological, mobility, spontaneity, and social), therapies (physiotherapy, respiratory therapy, ergotherapy, speech therapy, lymphatic drainage and psychological interventions), use of supporting aids (mobility aids, respiratory aids, home care aids, communication aids, tracheostomy and feeding tube), doctor visits, inpatient hospital and sleep clinic treatment, medication (riluzole, edaravone, antidepressants, non-opioid analgesic drugs, opioid analgesic drugs, benzodiazepines, cannabis, magnesium, vitamin d3, vitamin b and folic acid) and degree of care and support. The third part included the ALSAQ-5, the EQ-5D-5L and the revised ALS Functional Rating Scale (ALSFRS-R) questionnaires.

The ALSFRS-R is a well-established, reliable [3,41] and self-reported measurement that asks patients to assess their own functioning on a scale from 0 (unable to attempt the task) to 4 (normal function) on 12 items [42,43]. The total score ranges from 0 to 48, with 0 meaning total dependence and 48 meaning no impairment. The questions of the ALSFRS-R can be categorized into four domains: fine motor, gross motor, bulbar and respiratory function. It is the preferred clinical scale to measure disease severity in ALS [44]. 

The King’s Clinical Staging System (King’s stage) was derived from the ALSFRS-R [45]. The King’s stages are based on two realms: the number of affected body regions (bulbar, cervical, thoracic and lumbar) during disease progression and additional prognostic criteria [46,47]. The first three stages relate to the number of body regions displaying UMN and/or LMN signs (e.g., Stage 1 means that one region has signs). Stage 4 denotes the presence of prognostic criteria, with 4a corresponding to nutritional failure and 4b to respiratory failure. Stage 5 corresponds to death. 

Using a staging system is important because it is simple to understand and can help with resource management, as patients at different disease stages have different needs [45]. The King’s stages have been shown to correlate with the natural disease course and progression of ALS [48]. Balendra et al. showed that a majority of patients moved from one stage to the next, without skipping stages or moving backwards. Their study also confirmed a good correlation (92%) between the ALSFRS-R score and the King’s stages, though there remained some potential for over- or underestimation. Additionally, the King’s stages have been used in many previous clinical studies [5,12,46].

### 2.3. Health-Related Quality of Life Measures

The subjective, self-reported health state of the study participants was measured using the German versions of the ALSAQ-5 and the EQ-5D-5L. 

The EQ-5D-5L is a multifaceted questionnaire looking at five different dimensions of QoL: (1) mobility; (2) self-care; (3) usual activities; (4) pain/discomfort and (5) anxiety/depression. For each dimension, one single item is offered and can be answered on a scale ranging from “no problems” (level 1) to “extreme problems” (level 5) [49]. Answers to all five questions are accumulated and provide a unique five-digit number reflecting the patients’ self-reported state of health (e.g., 11111 if all questions were answered with level 1, or 55555 if all questions were answered with level 5) [32]. In order to translate the answers of the patients into a comparable measurement of QoL, the five-digit number is converted into an index value, ranging between −0.205 and 1.0 for Germany [49]. As part of the EQ-5D-5L, patients were also asked to rate their “health state today” on a visual analogue scale (EQ VAS) ranging from 0 to 100, with 100 being the best possible QoL score [32].

Following the instructions of the EQ-5D-5L User Manual [50], missing answers were coded with a value of 9 to allow for calculation of a total score and an index value. However, in relation to the comparison between the ALSAQ-5 and EQ-5D-5L questions (see below), these missing answers were excluded. As a result, the total n number of EQ-5D-5L index values differs from the *n* number of each individual question.

The ALSAQ-5 questionnaire measures the HRQoL in ALS patients by targeting disease-specific symptoms. It contains five questions with five possible answers ranging from “never” to “always” or “cannot do at all”, relating to the limb, bulbar and psychological areas: (1) physical mobility, (2) activities of daily living and independence, (3) eating and drinking, (4) communication and (5) emotional functioning. The value of using the ALSAQ-5 as opposed to the ALSAQ-40 is that it functions as an easy-to-use, bedside test to evaluate ALS-specific QoL. This has been found, unsurprisingly, to result in higher response rates, and consequently, more data [36]. 

The ALSAQ-5 yields a score ranging from 0 to 100, with 0 reflecting the best health state [51]. To calculate the percentage score of each individual question (i.e., Question 1 = x1, Question 2 = x2), we took the score provided (n), ranging from 1–5, divided it by 5, as there were five options, and then multiplied that number by 100, giving us the percentage. The formula for the individual question score is:

x1=(n÷5)×100.

In order to calculate the total ALSAQ-5 score, the mean of all five questions was calculated. If a patient did not fill in all five answers, he/she was excluded from the total score due to the diversity of the subject areas, which meant that the calculation of the HRQoL was not possible. Separately, we included data from each individual question answered, whether the patient had answered all of the other questions or not, for the purposes of the comparison with the EQ-5D-5L [51].

The two questionnaires can be easily compared because they both: (1) comprise of five questions; (2) offer five possible answers on a scale and (3) look at five different dimensions of QoL. Additionally, three of the questions (Q) overlap in their subject matter and can be directly compared. These are: Q1: mobility vs. (versus) physical mobility; Q2: self-care vs. activities of daily living and independence and Q5: anxiety/depression vs. emotional functioning. However, as the total score of the two questionnaires cannot be directly compared, because the EQ-5D-5L only calculates an overall score based on an index set specific to each country [49], the EQ VAS, which has been shown to correlate with the total EQ-5D-5L index value [5,52], was used in the comparison with the ALSAQ-5 total score. Both of these scales range from 0 to 100, though the “best” QoL scores in both are at opposite ends of the scale (ALSAQ-5 = 0 is best; EQ VAS = 100 is best). To allow for the alignment of the scales, not just numerically but with regards to the best possible score, at points, a reversed EQ VAS score (calculated by subtracting given the EQ VAS score from 100) was calculated, where 0 was defined as the best QoL.

As the EQ-5D-5L is widely used and has been validated for different (disease) cohorts for measurement of HRQoL, it provides a strong reference point for our analysis using the German version of ALSAQ-5, and thus we have compared the results of the two questionnaires in this study. The ALSAQ-5 has not been validated in German, though it has been validated in other European languages, for example in Dutch [53] and Italian [54]. Therefore, our study is a first step in showing whether there is a correlation between the two questionnaires in their German versions.

### 2.4. Statistical Analysis

Data management and analysis was completely performed at Hannover Medical School, Hannover, Germany. Statistical analysis was conducted using IBM^®^ Statistical Software Package of Social Science (SPSS^®^, Chicago, IL, USA) version 26. Frequency tables were used to determine the demographic data. The dependent variable (mean ALSAQ-5 total score) was assessed for normality using a quantile–quantile plot and a histogram. Due to most of the data points being situated along the diagonal line, the assumption of normality was not violated. Average differences of the mean ALSAQ-5 and EQ-5D-5L total scores were tested against the King’s stages (using a pairwise test) and ALS variant using a Student’s *t*-test, and equality of variance was determined by the Levene’s test. Although some variables deviated slightly from normality, the analysis was continued, based on the large sample size and the robustness of *t*-tests against violations of normality [55]. Furthermore, a Whitney–Mann U test was conducted between patients who had symptoms of respiratory insufficiency to assess the differences in QoL between patients who had been tracheostomized, had non-invasive ventilation, and had no ventilation at all. 

Regression analysis was performed in order to analyze the impact of the demographics and the different supporting aids and therapy forms on QoL and was done by individually testing possible variables (age, gender, BMI, marital status, currently working, genetics, first symptoms, ALSFRS-R total score, King’s stages, ambulatory status, care level, 24 h carer, professional support, house carer, family support, rehabilitation, visit to sleep clinic, insurance type, feeding tube, respiratory aids (tracheostomy, non-invasive ventilation and other respiratory aids), mobility aid, communication aid, caring aid, speech therapy, respiratory therapy, physiotherapy, ergotherapy, lymphatic drainage, psychological intervention, riluzole, antidepressants, opioid analgesics, non-opioid analgesics, benzodiazepines, antispasmodics, cannabis and edaravone) using a simple linear regression model. All significant variables were entered into a multiple regression model, which performed a backward selection in order to define the variables with the highest impact on QoL. The P–P plot suggested that the assumption of normality may have been violated, but due to the large sample size and the robustness of regression models against violations of normality, it is unlikely that a notable impact would have been made [56]. Furthermore, the residuals were shown to be independent of each other (Durbin–Watson = 2.247) and the constant based on the scatterplot showed no obvious signs of funneling.

In addition, all significant variables in the simple linear regression model were divided into subgroups (supporting aids, medication, therapies, care and others), which were also entered into different multiple linear regression models. In all models, the mean ALSAQ-5 total score was the dependent variable, whilst age and gender were always additionally included as independent variables. The ALSFRS-R score was included as a covariate in all models to ensure that the analysis took place relative to patient disease severity. King’s stages were not included in the multiple linear regression analysis as they have been shown to correlate with the ALSFRS-R [45,48].

A Spearman’s correlation was used to compare the results of the individual questions of the ALSAQ-5 and EQ-5D-5L. Internal consistency of the ALSAQ-5 questionnaire was measured using Cronbach’s alpha, which ranges on a scale from 0 to 1, with higher values denoting a greater internal consistency [57]. All statistical results should not be regarded as confirmatory, but rather as hypothesis generating. Due to the exploratory character of the study, we did not adjust for multiple testing.

The data are presented by providing the mean, median, standard deviation (SD), 95% confidence interval (CI), beta coefficients (β) and *p*-values. Individual missing values in different data and distinct questions resulted in differing *n* numbers. All *p*-values were two-tailed; a *p*-value of ≤0.05 was considered statistically significant.

## 3. Results

### 3.1. Participants

From 17 centers, 325 German ALS patients were included in the analysis for this study, whose detailed characteristics are presented in Table 1. The distribution of age, gender and first symptoms in our cohort showed to be representative according to the previous literature [3,10,58]. Regarding the aids, therapies and medications used, 68.2% of patients used a mobility aid. Mobility aids covered different aids relating to mobility, including, but not limited to, a walking stick, a walker and a wheelchair (both manual and electric). Concerning ventilation support, 20.3% had non-invasive ventilation, while only 4.3% of the described patients underwent tracheostomy. Of the patients, 52.0% had speech therapy, 31.8% used a communication aid and 19.9% took an antidepressant (see Table A1). Of the patients, 65.7% were classified as having care levels 3–5, representing a moderate to most severe loss of autonomy, similar to previous studies [5], whilst 14.9% had no level of care [59].

**Figure 2:**
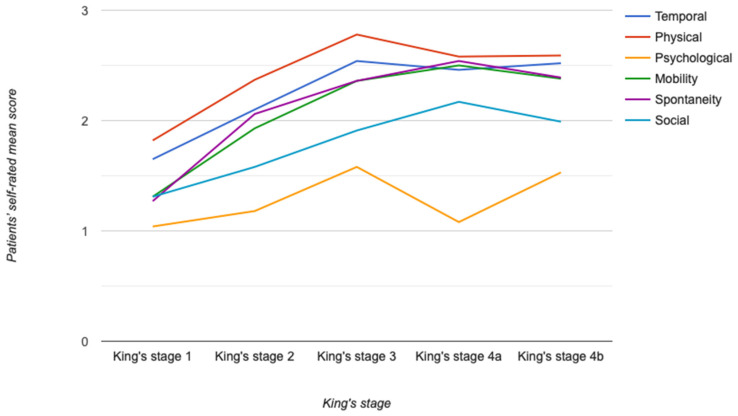
Chart showing the association of ALS with activities of daily living. Association between King’s stages (shown on the *x*-axis) and activities of daily living in different dimensions (with answers ranging from 0 = no impact to 3 = severe impact, shown on the *y*-axis). The temporal category relates to the patient’s self-reported impact of ALS on the time taken to do usual activities, as well as the impact on their free-time. Abbreviations: ALS, amyotrophic lateral sclerosis; King’s stage, King’s College Staging System.

In our cohort, increased disease severity according to the King’s stages was associated with higher impact on all activities of daily living (Figure 2). This association was evident up to and including King’s stage 3. However, for King’s stage 4a and 4b, the self-rated scores either plateaued or slightly decreased. Additionally, regardless of King’s stages, the mean scores in the psychological area, compared to the impact on the other areas, were consistently lower, suggesting less of an impact on daily activities.

### 3.2. Descriptive Analysis of HRQoL in the Total Cohort

The mean ALSAQ-5 total score was 56.93 (max. = 100, best QoL = 0). As shown in Table 1, our patients were impacted the least by eating and drinking (mean score 44.82), and the most in activities of daily living and independence (mean score 63.85). The mean EQ-5D-5L index value was 0.48 (max./best = 1.0). Having transposed the answers of the individual EQ-5D-5L questions to a scale of 0 to 100 (best = 0, analogous to the ALSAQ-5), our patients rated themselves as being most impacted in their usual activities (mean score 71.01), and least in anxiety/depression and pain/discomfort (mean scores 45.02 and 46.93). Finally, the mean EQ VAS score was 42.58 (max./best = 100).

**Figure 3:**
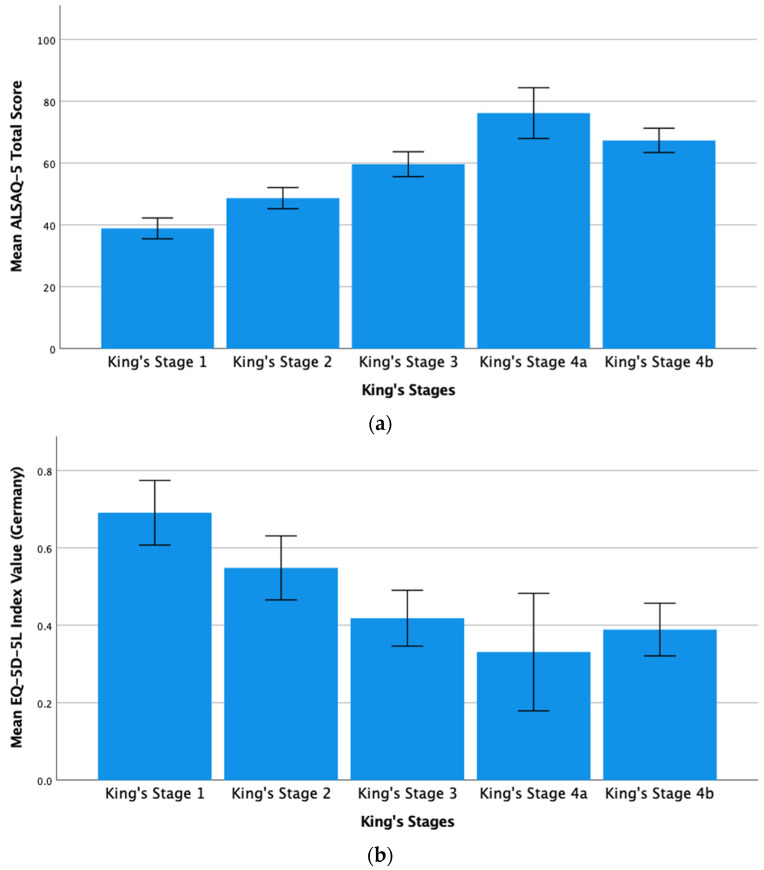
(**a**). Mean ALSAQ-5 total score on the basis of King’s stages. (**b**). Mean EQ-5D-5L index value (Germany) on the basis of King’s stages. Charts comparing the total mean scores of the ALSAQ-5 and EQ-5D-5L, according to their own scales, based on the King’s stages. For the ALSAQ-5 score, the higher score reflects a worse QoL, whereas for the EQ-5D-5L index value (Germany), the higher score reflects a better QoL. Accordingly, normality for King’s stage 4a was proven according to the Kolmogorov–Smirnov test. Error bars = 95% confidence interval. Significance levels, Figure 3a: *p* ≤ 0.01 (found after pairwise comparison between all King’s stages using the Student’s *t*-test). Significance levels, Figure 3b: *p* ≤ 0.05 (a statistically significant difference was found between King’s stage 1 and 2 compared with all other stages using the Student’s *t*-test in a pairwise comparison). Abbreviations: ALSAQ-5, Amyotrophic Lateral Sclerosis Assessment Questionnaire 5; EQ-5D-5L, EuroQol Five Dimension Five Level Scale; King’s stage, King’s College Staging System and QoL, quality of life.

Figure 3a,b illustrate that the HRQoL of our cohort decreased with increasing disease progression and King’s stage. As the mean total scores of the ALSAQ-5 and EQ-5D-5L were calculated differently, they were not directly compared statistically.

### 3.3. Positive Influence of Mobility Aids and Ventilator Support on HRQoL

Table 2 shows the results of the multiple linear regression analysis, which investigated the main influencing factors on HRQoL with the mean ALSAQ-5 total score as the dependent variable. The results showed that higher, and thus “better”, ALSFRS-R scores were associated with better QoL, in accordance with the current literature [5,9,10,11,60]. Accordingly, we included the ALSFRS-R as a covariate in further analyses in order to compare patients at similar disease progression with each other.

Moreover, the utilization of special aids was associated with better QoL. In particular, mobility aids and ventilator support (being tracheostomized and undergoing non-invasive ventilation) were associated with better QoL. Being tracheostomized even showed a greater beta coefficient than in previous studies [61,62,63]. It should be noted here that without the ALSFRS-R as a covariate, patients who had been tracheostomized had a much worse QoL than those who had not (mean ALSAQ-5 total score 85.09 vs. 55.81; best QoL = 0). Additionally, and again without the ALSFRS-R as a covariate, in an analysis of all patients who reported respiratory insufficiency, patients who had been tracheostomized had a significantly worse QoL compared to those who had non-invasive ventilation (*p* = 0.001, mean ALSAQ-5 total score 85.09 vs. 64.45), reflecting a previous study [64].

By contrast, regular intake of antidepressants, probably resulting from the symptoms or diagnosis of depression, was an independent predictor of reduced QoL. Additionally, congruent with previous literature [9], older age was also associated with worse QoL.

The significant therapies from the simple linear regression analysis were compared against one another in a separate multiple linear regression analysis, with the mean ALSAQ-5 total score as the dependent variable. We also included gender and age as other independent variables, and the ALSFRS-R total score as a covariate. In this model, speech therapy was found to be a negative predictor of QoL (β = 5.05, *p* < 0.001). Nevertheless, when comparing the QoL of patients with reduced bulbar functioning in relation to the use or non-use of speech therapy (without the ALSFRS-R as a covariate), no significant difference was found between the two groups.

By contrast, in further comparisons of the different types of care, aids and medications utilized by ALS patients, no additional impact in relation to QoL was observed.

### 3.4. Effect of Bulbar Dsyfunction on HRQoL

The ALSAQ-5 total scores showed that patients with *l*ALS reported a better QoL compared to those with *b*ALS (*b*ALS = 60.99 vs. *l*ALS 55.46, 0 = best QoL). Additionally, in the direct comparison of the individual questions of the two questionnaires, significant differences in QoL between *l*ALS and *b*ALS patients were observed (Table 3). In the ALSAQ-5, *l*ALS patients had better scores of QoL in questions relating to eating and drinking and communication, but consistently showed worse scores in questions relating to mobility and daily activities than *b*ALS patients. By contrast, in the EQ-5D-5L, *b*ALS patients reported better QoL than *l*ALS patients in every question, as well as in the EQ-5D-5L index value and the EQ VAS. Finally, in both questionnaires, there was no statistically significant difference whether a patient had *l*ALS or *b*ALS in relation to the psychological area.

### 3.5. Comparison of the EQ-5D-5L and the ALSAQ-5 Questionnaires

Table 4 shows the results of the Spearman’s correlation between the questions of the ALSAQ-5 and EQ-5D-5L. We observed a moderate correlation between Q1, Q2 and Q5 of both questionnaires. In addition, there was also a moderate correlation between the mean ALSAQ-5 total score and reversed EQ VAS score.

Furthermore, we tested the internal consistency of the ALSAQ-5 with a Cronbach’s alpha presenting at an acceptable internal consistency of 0.722. As shorter test lengths reduce Cronbach’s alpha [57], the internal consistency of the ALSAQ-5 should be given greater weight considering it consists of only five questions. 

## 4. Discussion

To date, our study investigated the largest ALS patient cohort in Germany with regards to different impact factors on QoL, including supporting aids and therapy forms. The results showed that mobility aids, non-invasive ventilation and tracheostomy significantly improved QoL, when the ALSFRS-R was included as a covariate, whereas speech therapy and antidepressant medication, which could be associated with dysarthria/*b*ALS and depression, respectively, were markers for significantly worse QoL. Other factors that had a significant impact on QoL were disease progression (further progression led to worse QoL) and age (increased age led to worse QoL). In a direct comparison, the results of the ALSAQ-5 showed that *b*ALS patients had a worse QoL than *l*ALS patients. Moreover, we showed that the ALSAQ-5 was a valid instrument to measure HRQoL in ALS patients.

The intervention that had the most significant influence on improving patient QoL was the use of a mobility aid. Lack of mobility significantly lowered the QoL, as shown by our results and by several previous studies [9,42,66]. This finding is important as it confirms that a mobility aid can directly counter the negative effects of the deterioration of mobility on QoL. As two thirds of ALS patients have *l*ALS, with severe and rapidly progressive muscle weakness early in the disease course [4], a mobility aid can provide important benefits for the majority of ALS patients and should be made available as early as possible. Interestingly, though, this result is in contrast with a previous study that found that the use of a wheelchair reduced QoL [5]. However, the different result could be due to the fact that our study looked at the broader category of a mobility aid, which included, but was not limited to, the use of a wheelchair. In addition, being ambulatory was not found to have a significant positive impact on QoL in our study cohort. Thus, mobility in general (i.e., being able to move independently from point A to point B by any means and maybe not necessarily being able to walk between them) seems to play a central role for ALS patients and their QoL.

Furthermore, the ALSFRS-R was used as a covariate in the multiple linear regression analysis in order to compare patients relative to their disease severity. We found that the inclusion of the ALSFRS-R, and therefore disease severity, greatly influenced the results of the other independent variables.

The results of our multiple linear regression model found that being tracheostomized had a positive influence on QoL despite the small number of eligible participants (*n* = 14) in our cohort. Nevertheless, it should be noted that when patients who had been tracheostomized were compared to those who had not, independent of the ALSFRS-R total score, they had a far reduced QoL. Additionally, in a comparison of patients who had respiratory insufficiency, patients who had been tracheostomized had a significantly reduced QoL compared to those who had non-invasive ventilation, reflecting a previous study [64], and those requiring no ventilation, that again reflected their disease progression.

Tracheostomy, despite the procedure-associated risks and the potential of patients to reach a “locked-in” state, has previously been found to result in acceptable QoL [61,62,63] and increased survival in ALS [67]. Additionally, a Cochrane review noted that while tracheostomy prolongs life, it does not necessarily improve QoL, again comparing it with non-invasive ventilation [68]. As can be taken from these conflicting findings, more research is needed to understand the implications and impact of being tracheostomized on QoL.

The use of non-invasive ventilation was also found to significantly improve QoL. This finding is in line with previous studies, which have found non-invasive ventilation to be efficient at treating respiratory failure, prolonging life and also improving QoL [15,30,69,70,71,72,73]. Corresponding to our findings regarding tracheostomy and non-invasive ventilation, another previous study found that ventilated patients had a better QoL than non-ventilated patients, potentially due to the beneficial effects of ventilation on lessening daytime sleepiness, which supports our results [74]. As a consequence, patients will, indisputably, benefit from the access to ventilation support. 

In our analysis of the therapies used by ALS patients, our results showed that receiving speech therapy was a predictor of worse QoL. Körner et al. [19] noted that while speech therapy can be useful during early disease stages, the decline in speech function could only be slightly delayed by speech therapy. They ultimately found in their direct comparison of a communication aid and speech therapy in ALS patients with dysarthria and anarthria, that speech therapy was not as effective in improving QoL compared to a communication aid. In line with this finding, the results of our analysis of patients with reduced bulbar functioning showed no significant difference whether the patient had undergone speech therapy or not, suggesting that this intervention is an inadequate therapy form to delay this decline in bulbar function. Some further studies proved the impact of communication devices on QoL. For example, the Tobii Dynavox has been found to have a strong impact on QoL for patients who are being ventilated, mostly due to the effect it has on their autonomy [24]. However, in contrast to the literature noted above, our results relating to the use of a communication aid did not show a significant influence on QoL.

In looking further at the distinction between *l*ALS and *b*ALS patients, we found that patients with the *l*ALS variant had a significantly better QoL than those with *b*ALS as measured by the ALSAQ-5. This finding concurs with previous studies that found *b*ALS to be associated with a poorer prognosis and QoL [3,27,28,29,30,31], and a shorter survival time [3,27,58]. This was also supported by the finding that, in this study, anticholinergic drugs were found to be a negative predictor of QoL, as this specific treatment was associated with *b*ALS more often. Nevertheless, it has been previously shown that alleviation of drooling had a positive impact on QoL [23], so this treatment should be offered to patients, if appropriate. Besides, further research and resources should be put into developing additional effective interventions for *b*ALS patients in particular.

An easy-to-use, bed-side test to evaluate ALS-specific HRQoL during clinical routine will allow for more focused and individually relevant treatment for patients and also contribute to more ALS-specific data [66]. While the ALSAQ-40 has been used more widely in ALS studies [75,76], answering 40 questions is a burden on patients, which compromises data collection [36,53]. To this end, the ALSAQ-5 was developed and has been validated in multiple languages [36,53,54], but not yet in German. While the ALSAQ-5 has been validated with the longer version ALSAQ-40, it has not been compared to the more established, but also more general HRQoL questionnaire, the EQ-5D-5L. 

Having found a worse QoL in *b*ALS patients in the ALSAQ-5 total score, we were surprised to note that the opposite was true according to the EQ-5D-5L index score (Germany). While the ALSAQ-5 has two questions relating to *l*ALS symptoms (Q1 and Q2) and two questions relating to *b*ALS symptoms (Q3 and Q4), the EQ-5D-5L does not have any questions relevant to the specific symptoms of *b*ALS patients, with Q1-Q4 focusing on *l*ALS or general symptoms or factors. It is a natural consequence, then, that *b*ALS patients showed a better overall QoL in the EQ-5D-5L index value and in the individual EQ-5D-5L questions. Nevertheless, while the overall QoL was worse in *b*ALS patients according to the ALSAQ-5 total score, it was also shown that *b*ALS patients performed better (i.e., had a better QoL) in Q1 and Q2 of the ALSAQ-5 questionnaire compared to *l*ALS patients. This highlights the need for a balanced HRQoL assessment in clinical practice that covers both *l*ALS and *b*ALS symptoms, which our study showed the ALSAQ-5 to be.

Building on this, a strong Spearman rho correlation and statistical significance was found between Q1 and Q2 of the two questionnaires. Furthermore, regarding Q3 and Q4, a weak correlation was found between Q3, while no correlation was found between Q4. The weak correlation between Q3 could result from the slightly similar nature of the question (eating and drinking vs. usual activities), as eating and drinking can be seen as part of one’s usual activities. However, this broader category of usual activities does not necessarily encompass the specific bulbar symptom that reduces the patient’s ability to eat or drink. These results reinforce our hypothesis that the ALSAQ-5 is a more specific questionnaire for ALS patients, providing a more accurate view of HRQoL in ALS than the EQ-5D-5L. A moderate Spearman rho correlation was found between Q5 of the two questionnaires, which relates to the psychological area, which can affect both *l*ALS and *b*ALS without distinction. Moreover, the analysis of the Cronbach’s alpha of the ALSAQ-5 showed an acceptable internal consistency, especially considering the shortness of the questionnaire [57]. Our results, therefore, have not only shown that the ALSAQ-5 is a reliable questionnaire and can be of use in clinical practice, but that the EQ-5D-5L is not sufficiently equipped to accurately assess the HRQoL of patients with ALS.

The results of our study relating to psychological parameters were inconsistent. Previous studies have found that there is a clear association between anxiety and depression and worse QoL in ALS [10,74,77,78,79]. However, our results showed that regardless of the King’s stages, patients reported to be least affected psychologically, compared to the impact on the other areas of daily living. This could also be seen in patients’ answers to individual questions of the EQ-5D-5L questionnaire, for example regarding anxiety/depression (Q5), which, with pain/discomfort (Q4), showed a better QoL than seen in the other dimensions. Conversely, in the results of the ALSAQ-5, emotional functioning (Q5) followed closely behind physical mobility (Q1) and activities of daily living and independence (Q2), in having a negative impact on QoL. The inconsistency of our data relating to the psychological impact of ALS on patients matches previous findings [7,9,79,80]. One possible explanation for the difficulty in having consistent data with regards to psychological factors is that the data comes from questionnaire scores, which have been found to be “insufficient for a psychiatric diagnosis” [77]. Moreover, it has been reported that depression correlates with reduced QoL in ALS. However, depression decreases during the disease course if the patient has the chance to cope with the disease, leading to an increased QoL in later disease stages [74]. Besides education, coping and QoL are also positively influenced by spirituality and religion [25,26,74].

In addition, we observed that patients who did not use antidepressants had a significantly better QoL, whereas it is known that up to 50% of ALS patients are symptomatically treated with antidepressants [81]. Therefore, our results support the above-mentioned association between depression and worse QoL in ALS. However, antidepressants are also prescribed to tackle other ALS-related symptoms, such as uncontrollable laughing and crying [22] or excessive salivation [82]. One study found that patients had a higher risk of depression in the year before and the year after ALS diagnosis. Depression, as hypothesized in that study, could also be an early manifestation of frontal lobe degeneration and thereby imply a faster disease progression [82]. Additionally, a pseudobulbar affect could also be misdiagnosed as a depressive symptom by patients’ carers, leading to the suspicion of reduced QoL [83]. Nevertheless, and similar to the assessment of QoL, accurate and validated questionnaires need to be used to collect further data in this area, as has already been done by some authors [74]. Though desirable, it might be quite difficult to analyze the complexity of all factors that might impact an ALS patient’s QoL in one study, but further longitudinal investigations of large cohorts may help to address further relevant questions.

### Limitations and Strengths

One of the limitations of this study is the collection of self-reported primary patient data. With regard to relevant interventions and therapies, we thus were only able to analyze factors that the patients themselves stated to use. This meant, for example, that medication such as Nuedexta, which has been previously shown to improve QoL, was not assessed because none of our patients were taking it. Therefore, the scope of analyzing interventions as predictors of QoL may be limited and we only discussed some of the factors that impact a patient’s QoL. However, the advantage of a self-reported approach is that patients are given the possibility to name individual influencing factors in context of their daily living. Nevertheless, in order to account for all available interventions, a comprehensive survey based on a predefined list would be necessary. Another limitation is the cross-sectional study design, which does not allow a depiction of the full picture of the patients over the disease course. However, a strength of cross-sectional studies is that they remove the possibility of floor- or ceiling-effects (i.e., where the majority of patients either score in the lower or upper boundaries of the questionnaire [11]). Another limitation lies in a potential bias in how the 17 centers decided on how to distribute the questionnaires and handle the follow-up. Moreover, as patients were recruited at specialized ALS-centers, we cannot exclude a selection bias towards (1) less severely affected patients who were still able to travel long distances to visit these centers and (2) more motivated, and potentially more educated or less cognitively impaired, patients who were willing to participate in clinical studies. Additionally, the socioeconomic status of patients, which has been found to be a big determinant of their ability to cope with the challenges that ALS poses and directly correlates with their QoL, was unfortunately not captured in our questionnaire.

On the other hand, a strength of this study was that the study sample was, with the exception of King’s stage 4a, about evenly split between the other King’s stages, which allowed us to draw a rather detailed picture on QoL throughout the different stages within disease progression. In addition, our participants were also evenly split across the different German states corresponding to the general population distribution in Germany (see Table A2). Finally, the main strength of our findings above lies in our large sample size, which included *n* = 325 patients from all over Germany, making our study generalizable, especially regarding the gender distribution, ALS variant and disease progression.

## 5. Conclusions

All in all, this study shows a high impact of ALS and its progression on individual HRQoL. Therefore, possible future therapies that attempt to stop or delay disease progression have a greater potential to lower the individual disease burden and increase QoL. The early and individually appropriate supply with special aids to maintain the best possible independent mobility should be one focus of clinical ALS management. Moreover, respiration significantly affects QoL. Therefore, information on the effect of respiratory aids on QoL should be provided to patients and taken into account in the further informed decision processes. Furthermore, greater access to ventilation support should be made available to patients who need it so they can benefit from it. Finally, the ALSAQ-5, an easy-to-use, bedside test, is more appropriate than the EQ-5D-5L to be applied to ALS patients as it specifically allows for self-assessment of bulbar symptoms.

## Figures and Tables

**Table 1:** Characteristics of the ALS patient cohort and the mean overall and individual question results of the quality of life (QoL) questionnaires, with *n* representing the number of patients who answered the specific question. The bold text refers to the heading of the question, whilst the roman text represents the subgroups.

Parameter	*n* (Percentage)	Mean (SD)	Median (Range)
**Age**	**325**	**63.94 (11.42)**	**64 (27–88)**
**Gender**	**325**		
male	203 (62.5%)		
female	122 (37.5%)		
**BMI (kg/m^2^)**	**323**	**24.15 (4.40)**	**24.21 (10.57–39.18)**
**Marital status**	**324**		
single	40 (12.3%)		
with partner	284 (87.7%)		
**Currently working**	**306**		
yes	51 (16.7%)		
no	255 (83.3%)		
**Genetics**	**312**		
familial ALS	14 (4.5%)		
sporadic ALS	298 (95.5%)		
**First symptoms**	**287** ^1^		
bulbar (*b*ALS)	85 (29.6%)		
limb (*l*ALS) ^2^	202 (70.4%)		
**ALSFRS-R total score (max./best = 48)**	**325**	**30.92 (10.29)**	**33 (1–48)**
**King’s stages**	**325**		
stage 1	57 (17.5%)		
stage 2	70 (21.5%)		
stage 3	83 (25.5%)		
stage 4a	24 (7.4%)		
stage 4b	91 (28.0%)		
**Ambulatory**	**324**		
Yes ^3^	239 (73.8%)		
no	85 (26.2%)		
**Care level** ^4^	**268**		
none	40 (14.9%)		
level 1	7 (2.6%)		
level 2	45 (16.8%)		
level 3	79 (29.5%)		
level 4	56 (20.9%)		
level 5	41 (15.3%)		
**24 h carer**	**303**		
yes	122 (40.3%)		
no	181 (59.7%)		
**ALSAQ-5 total score (max. = 100, best = 0)**	**288**	**56.93 (19.46)**	**56 (20–100)**
Q1: physical mobility (max. = 100, best = 0)	294	60.41 (28.19)	60 (20–100)
Q2: activities of daily living and independence (max. = 100, best = 0)	296	63.85 (27.61)	80 (20–100)
Q3: eating and drinking (max. = 100, best = 0)	299	44.82 (29.19)	40 (20–100)
Q4: communication (max. = 100, best = 0)	300	55.60 (31.50)	60 (20–100)
Q5: emotional functioning (max. = 100, best = 0)	298	59.80 (25.24)	60 (20–100)
**EQ-5D-5L index value (max./best = 1)**	**304**	**0.48 (0.34)**	**0.52 (−0.205–1)**
Q1: mobility (max. = 100, best = 0) ^5^	307	67.30 (29.26)	80 (20–100)
Q2: self-care (max. = 100, best = 0)	308	64.35 (30.08)	60 (20–100)
Q3: usual activities (max. = 100, best = 0)	307	71.01 (26.02)	80 (20–100)
Q4: pain/discomfort (max. = 100, best = 0)	306	46.93 (22.24)	40 (20–100)
Q5: anxiety/depression (max. = 100, best = 0)	307	45.02 (21.80)	40 (20–100)
**EQ VAS total score (max./best = 100)**	**304**	**42.58 (24.36)**	**40.00 (0–95)**

Abbreviations: ALS, amyotrophic lateral sclerosis; ALSAQ-5, Amyotrophic Lateral Sclerosis Assessment Questionnaire 5; ALSFRS-R, Revised Amyotrophic Lateral Sclerosis Functional Rating Scale; *b*ALS, “bulbar-onset” ALS; BMI, body mass index; EQ-5D-5L, EuroQol Five Dimension Five Level Scale; EQ VAS, EuroQol Visual Analogue Scale; King’s stage, King’s College Staging System; *l*ALS, “limb-onset” ALS; max., maximum; *n*, number; Q, question; SD, standard deviation. ^1^ *n* = 287 due to missing answers or patients manifesting other first symptoms. ^2^ Of those patients diagnosed with first limb symptoms, 55 (27.2%) had upper limb symptoms, 81 (40.1%) had lower limb symptoms, 17 (8.4%) had fasciculations and cramps and 49 (24.3%) had unspecified muscle weakness. ^3^ Including being ambulatory with the help of an aid. ^4^ Patients were classified according to German statutory care insurance levels (as updated by the state in 2017). Higher levels of care correspond to greater loss of autonomy (level 1 = minor impairment of individual autonomy; level 5 = severe impairment of individual autonomy with special care requirements), also see [5,59]. ^5^ To calculate the percentage score of each individual question of the EQ-5D-5L, we took the score provided (*n*), ranging from 1–5, divided it by 5, as there were five options, and then multiplied that number by 100 to give us our percentage.

**Table 2:** The final multiple linear regression model that showed the influencing factors on QoL in ALS, with the ALSAQ-5 total score being the dependent variable. It included variables that were statistically significant in the simple linear regression model (*p*-value ≤ 0.05) and were analyzed in a multiple regression model, which performed a backward selection. The results are arranged by *p*-values. The maximum *n* included in the analysis was 288, as this equaled the total number of fully answered ALSAQ-5 questionnaires. Abbreviations: ALS, amyotrophic lateral sclerosis; ALSAQ-5, Amyotrophic Lateral Sclerosis Assessment Questionnaire 5; ALSFRS-R, Revised Amyotrophic Lateral Sclerosis Functional Rating Scale; QoL, quality of life; Std. Error, Standard Error.

Parameter	Beta Coefficient (β)	Std. Error	t	*p*-Value	95% Confidence Interval	
					Lower Margin	Upper Margin
ALSFRS-R total score	−1.96	0.157	−12.461	<0.001	−2.271	−1.649
Mobility aid = yes	−7.60	2.219	−3.424	0.001	−11.985	−3.211
Tracheostomy = yes	−14.80	5.046	−2.932	0.004	−24.770	−4.820
Antidepressants = yes	5.95	2.162	2.754	0.007	1.679	10.227
Age	0.18	0.080	2.294	0.023	0.025	0.343
Non-invasive ventilation = yes	−5.71	2.614	−2.186	0.030	−10.880	−0.546
House helper = yes	−3.37	1.841	−1.831	0.069	−7.012	0.268
Rehabilitation = yes	3.51	2.008	1.747	0.083	−0.460	7.477
Opioid analgesic drugs = yes	6.87	4.002	1.716	0.088	−1.042	14.782
Ergotherapy = yes	−3.08	1.859	−1.659	0.099	−6.758	0.591
Currently working = yes	3.89	2.635	1.475	0.142	−1.322	9.095
Ambulatory = yes	3.99	2.809	1.420	0.158	−1.563	9.542
Non-opioid analgesic drugs = yes	−4.12	2.993	−1.375	0.171	−10.033	1.801

**Table 3:** Comparison of the difference in QoL (both overall and specifically relating to the different dimensions of the questionnaires) between *l*ALS and *b*ALS patients based on the total mean scores and individual question scores of the ALSAQ-5 and EQ-5D-5L questionnaires, with *n* representing the number of patients who answered the specific question. In order to directly compare the results of the individual questions of the two questionnaires, the individual question scores of the EQ-5D-5L (ranging from 1–5), were converted, by taking the score provided, ranging from 1–5, dividing it by 5, as there were five options, and then multiplying that number by 100, to a scale from 0–100, with 0 = best QoL. Effect sizes of r = 0.50, r = 0.30, and r = 0.10 served as thresholds for large, medium and small effects [65], respectively. Abbreviations: ALSAQ-5, Amyotrophic Lateral Sclerosis Assessment Questionnaire 5; *b*ALS, “bulbar-onset” ALS; df, degrees of freedom; EQ-5D-5L, EuroQol Five Dimension Five Level Scale; EQ VAS, EuroQol Visual Analogue Scale; *l*ALS, “limb-onset” ALS; max., maximum, *n*, number; QoL, quality of life; SD, standard deviation; t, standard error.

Parameter	*l*ALS	*b*ALS	*l*ALS vs. *b*ALS
**ALSAQ-5 total score (max. 100, best = 0)**			
*n*	179	77	
mean (SD)	55.46 (19.76)	60.99 (19.23)	t = −2.07; df = 254; *p* = 0.040; r = 0.13
median (range)	56 (20–100)	60 (20–100)	
**Q1: Physical mobility (max. 100, best = 0)**			
*n*	183	78	
mean (SD)	63.39 (27.41)	50.26 (28.96)	t = 3.48; df = 259; *p* = 0.001; r = 0.21
median (range)	60 (20–100)	40 (20–100)	
**Q2: Activities of daily living and independence (max. 100, best = 0)**			
*n*	186	77	
mean (SD)	69.78 (25.40)	48.05 (27.20)	t = 6.18; df = 261; *p* < 0.001; r = 0.36
median (range)	80 (20–100)	40 (20–100)	
**Q3: Eating and drinking (max. 100, best = 0)**			
*n*	186	80	
mean (SD)	39.57 (26.79)	60.50 (31.50)	t = −5.19; df = 130.41; *p* < 0.001; r = 0.41
median (range)	20 (20–100)	60 (20–100)	
**Q4: Communication (max. 100, best = 0)**			
*n*	187	80	
mean (SD)	45.45 (28.25)	84.75 (19.16)	t = −13.20; df = 215.23; *p* < 0.001; r = 0.67
median (range)	40 (20–100)	100 (20–100)	
**Q5: Emotional functioning (max. 100, best = 0)**			
*n*	186	80	
mean (SD)	57.96 (25.43)	62 (25.77)	t = −1.18; df = 264; *p* = 0.237; r = 0.07
median (range)	60 (20–100)	60 (20–100)	
**EQ-5D-5L index value (max./best = 1)**			
*n*	191	79	
mean (SD)	0.42 (0.32)	0.66 (0.33)	t = −5.43; df = 268; *p* < 0.001; r = 0.31
median (range)	0.43 (−0.21–1)	0.81 (−0.14–1)	
**Q1: Mobility (max. 100, best = 0)**			
*n*	192	81	
mean (SD)	72.92 (26.60)	52.35 (30.10)	t = 5.34; df = 135.28; *p* < 0.001; r = 0.42
median (range)	80 (20–100)	40 (20–100)	
**Q2: Self-care (max. 100, best = 0)**			
*n*	191	82	
mean (SD)	71.20 (27.42)	47.07 (30.69)	t = 6.43; df = 271; *p* < 0.001; r = 0.36
median (range)	80 (20–100)	40 (20–100)	
**Q3: Usual activities (max. 100, best = 0)**			
*n*	192	81	
mean (SD)	76.04 (23.36)	57.04 (28.30)	t = 5.33; df = 128.14; *p* < 0.001; r = 0.43
median (range)	80 (20–100)	60 (20–100)	
**Q4: Pain/Discomfort (max. 100, best = 0)**			
*n*	192	80	
mean (SD)	49.27 (22.78)	40.50 (19.87)	t = 3.17; df = 168.35; *p* = 0.002; r = 0.24
median (range)	40 (20–100)	40 (20–100)	
**Q5: Anxiety/Depression (max. 100, best = 0)**			
*n*	192	81	
mean (SD)	46.25 (22.29)	40.74 (21.32)	t = 1.89; df = 271; *p* = 0.060; r = 0.11
median (range)	40 (20–100)	40 (20–100)	
**EQ VAS total score (max./best 100)**			
*n*	190	80	
mean (SD)	39.72 (24.18)	50.99 (24.40)	t = −3.49; df = 268; *p* = 0.001; r = 0.21
median (range)	40 (0–95)	50 (0–90)	

**Table 4:** Spearman’s rho correlation between the individual questions of the ALSAQ-5 and EQ-5D-5L, and the ALSAQ-5 total score and the reversed EQ VAS score, with *n* representing the number of patients who answered both questions. Abbreviations: ρ (rho), Spearman’s rho; ALSAQ-5, Amyotrophic Lateral Sclerosis Assessment Questionnaire 5; AQ1–5, Amyotrophic Lateral Sclerosis Assessment Questionnaire Questions 1–5; EQ1–5, EuroQol Five Dimension Five Level Scale Questions 1–5; EQ VAS, EuroQol Visual Analogue Scale; *n*, number; QoL, quality of life; vs., versus.

Comparison	ρ (rho)	*p*-Value	*n*
AQ1 vs. EQ1	0.73	<0.001	290
AQ2 vs. EQ2	0.71	<0.001	292
AQ3 vs. EQ3	0.27	<0.001	295
AQ4 vs. EQ4	−0.01	0.878	295
AQ5 vs. EQ5	0.55	<0.001	294
ALSAQ-5 total score vs. reversed ^1^ EQ VAS score	0.62	<0.001	283

^1^ Both the EQ VAS and the ALSAQ-5 range on a scale from 0–100, though the “best” QoL scores in both are at opposite ends of the scale (ALSAQ-5 = 0 is best; EQ VAS = 100 is best). To allow for the alignment of the scales, not just numerically but with regards to the best possible score, a reversed EQ VAS score (by subtracting the given EQ VAS score from 100) was calculated, where 0 = best QoL.

## Data Availability

The data presented in this study are available on request from the corresponding author. The data are not publicly available due to privacy and ethical reasons.

## Appendix A

**Table A1:** Patient characteristics: aids, therapies and medications. Table showing the number of different patients using the various aids, therapies and medications, including tracheostomy, non-invasive ventilation and a mobility aid. Abbreviations: *n*, number.

Parameter	*n* (Percentage)
Feeding tube	325
Yes	44 (13.5%)
No	281 (86.5%)
Respiratory aid	317
Yes	101 (31.9%)
No	216 (68.1%)
Tracheostomy	325
Yes	14 (4.3%)
No	311 (95.7%)
Non-invasive ventilation	325
Yes	66 (20.3%)
No	259 (79.7%)
Other respiratory aid (incl. portable oxygen device, inhalation device and suction device)	317
Yes	21 (6.6%)
No	296 (93.4%)
Mobility aid	321
Yes	219 (68.2%)
No	102 (31.8%)
Communication aid	314
Yes	100 (31.8%)
No	214 (68.2%)
Caring aid	317
Yes	186 (58.7%)
No	131 (41.3%)
Speech therapy	325
Yes	169 (52.0%)
No	156 (48.0%)
Respiratory therapy	325
Yes	31 (9.5%)
No	294 (90.5)
Physiotherapy	325
Yes	261 (80.3%)
No	64 (19.7%)
Ergotherapy	325
Yes	178 (54.8%)
No	147 (45.2%)
Lymphatic drainage	325
Yes	14 (4.3%)
No	311 (95.7%)
Psychological intervention	299
Yes	29 (9.7%)
No	270 (90.3%)
Riluzole	321
Yes	275 (85.7%)
No	46 (14.3%)
Antidepressants	321
Yes	64 (19.9%)
No	257 (80.1%)
Opioid analgesic drugs	321
Yes	16 (5.0%)
No	305 (95.0%)
Non-opioid analgesic drugs	321
Yes	27 (8.4%)
No	294 (91.6%)
Benzodiazepines	321
Yes	10 (3.1%)
No	311 (96.9%)
Antispasmodics	321
Yes	33 (10.3%)
No	288 (89.7%)
Anticholinergics	325
Yes	29 (8.9%)
No	296 (91.1%)
Cannabis ^1^	321
Yes	11 (3.4%)
No	310 (96.6%)
Edaravone	321
Yes	20 (6.2%)
No	301 (93.8%)

^1^ No further information is available on the type or use of cannabis, as this data was collected through an open question that asked patients to list the other medication that they are on.

**Table A2:** Regional distribution of ALS patients included in this study compared with the regional distribution by state of the general population in Germany. The distribution of our patients roughly matches the regional population distribution by state in Germany. Due to rounding, percent do not add up to exactly 100. Abbreviations: ALS, amyotrophic lateral sclerosis; *n*, number.

State	*n* Included in Study (Percentage)	Total Population of the State, 2019 [in Thousands] (Percentage) [84]
Lower Saxony	133 (40.9%)	7994 (9.7%)
North Rhine-Westphalia	74 (22.8%)	17,947 (21.6%)
Bavaria	52 (16.0%)	13,125 (15.8%)
Saxony	15 (4.6%)	4072 (4.9%)
Baden-Wuerttemberg	10 (3.1%)	11,100 (13.3%)
Hesse	8 (2.5%)	6288 (7.6%)
Schleswig-Holstein	8 (2.5%)	2904 (3.5%)
Saxony-Anhalt	8 (2.5%)	2195 (2.6%)
Rhineland-Palatinate	4 (1.2%)	4094 (4.9%)
Bremen	4 (1.2%)	681 (0.8%)
Mecklenburg-Western Pomerania	3 (0.9%)	1608 (1.9%)
Brandenburg	2 (0.6%)	2522 (3.0%)
Hamburg	2 (0.6%)	1847 (2.2%)
Thuringia	1 (0.3%)	2133 (2.6%)
Berlin	0 (0.0%)	3669 (4.4%)
Saarland	0 (0.0%)	987 (1.2%)
(Did not answer)	1 (0.3%)	N/A
